# Branching Processes: Their Role in Epidemiology

**DOI:** 10.3390/ijerph7031204

**Published:** 2010-03-19

**Authors:** Christine Jacob

**Affiliations:** National Agricultural Research Institute, UR341, Department of Applied Mathematics and Informatics, F-78352 Jouy-en-Josas, France; E-Mail: christine.jacob@jouy.inra.fr

**Keywords:** branching process, age-dependence, population-dependence, extinction time, epidemic size

## Abstract

Branching processes are stochastic individual-based processes leading consequently to a bottom-up approach. In addition, since the state variables are random integer variables (representing population sizes), the extinction occurs at random finite time on the extinction set, thus leading to fine and realistic predictions. Starting from the simplest and well-known single-type Bienaymé-Galton-Watson branching process that was used by several authors for approximating the beginning of an epidemic, we then present a general branching model with age and population dependent individual transitions. However contrary to the classical Bienaymé-Galton-Watson or asymptotically Bienaymé-Galton-Watson setting, where the asymptotic behavior of the process, as time tends to infinity, is well understood, the asymptotic behavior of this general process is a new question. Here we give some solutions for dealing with this problem depending on whether the initial population size is large or small, and whether the disease is rare or non-rare when the initial population size is large.

## 1. Introduction

Mathematical models of propagation of a disease in given populations play a central role for understanding this propagation, for predicting the future extension of the outbreak, its extinction time, and for evaluating the efficiency of control measures. Of course the validity and the richness of results of a model strongly depend on the reliability and the accuracy of the model. A fine predictive model should be built as far as possible in a rigorous mechanistic way starting from the mechanism of exposure/infection of each individual and taking into account their variability. The population dynamic described by births, deaths, migrations should also be taken into account, especially when the incubation time is relatively long in comparison with this dynamic. Of course, the time unit of the model should be chosen in keeping with the respective durations of each health state and the data.

Nevertheless, since the asymptotic behavior, as time tends to infinity, of deterministic models in continuous time are more easily studied than that of discrete time models or stochastic models, a large literature in applied mathematical journals is devoted to such theoretical studies [19], while for statistical purposes, either descriptive non dynamical models or very simple stochastic models are used, such as a chain binomial model for the propagation of *SIS* or *SIR* diseases in closed populations [5, 6], or BGW (Bienaymé-Galton-Watson) branching processes on the clinical (or diagnosed) cases as an approximate model of the beginning of an outbreak [2, 3, 4, 6, 10, 11, 20]. These two approaches are both individual-based (each individual transition is given) and stochastic (individual variability is taken into account) but the first one takes into account the individual health state evolution *S* → *I* → *S* or *S* → *I* → *R*, where *S* means susceptible, *I* means infective or clinical cases (according to authors and purposes) and *R* means removed from the susceptible population, either by immunization or death; while the second approach directly models the process of *I* individuals, without analyzing this mechanism of exposure/infection. The behavior of these two types of processes are well-known. The chain binomial models are just Markov chains in a finite state space, and since there is no immigration and no incubation period, the state “0 infected individual” is an absorbing state. Concerning BGW processes, a huge literature exists for describing their properties. In fact these two different types of models may be written as particular cases of a large class of branching processes with individual transitions that may be population and age dependent, and which can take into account both the population dynamics and the disease dynamic.

Branching processes were initiated in the nineteen century by Bienaymé and then by Galton and Watson, for studying the extinction of some family names. Since this time, the complexity of these processes continues to increase allowing to describe more and more realistic population dynamics. These processes are based on the simple property that the population size of each considered type (such as clinical cases here) at each time is calculated as the sum of all the new individuals (“offspring”) of this type who are generated by the individuals of the population at the previous times. Since the modelled variables are integers, then the extinction time of the population of each type is finite on the set of trajectories which extinct. This is a finer and more realistic property than the asymptotic extinction time given by a deterministic model. Since the population dynamic may influence the disease dynamic, and conversely, these two dynamics should be explicitly taken into account in the model, thus leading to rigorous, but not simple, multitype models where each type should represent an health state crossed with influence factors levels. Typical examples of such influence factors are age, geographical locations. However, some authors model directly the time evolution of the incidence of clinical cases, without explicating all the intermediary steps.

In the following subsections, we present such direct models starting from the simplest one, the single-type Bienaymé-Galton-Watson process, and finishing by a general and rigorous approach that takes into account the intermediary steps and the population dynamic, and is based on age-dependent and population-dependent individual transitions. We focus on models in discrete time since they have the double advantage to be easily written as recursive models and to easily correspond to the time unit of observation, which offers a pedagogic framework and moreover facilitates the model validation and the estimation of unknown parameters. We study here the behavior of these models. From now on, all results are given conditionally to the initial value *F*_0_ of the process and the notation “|*F*_0_” will be therefore omitted for the sake of simplicity of formulas. The proofs are given in detail in [23] and will be omitted here.

## 2. Single-type Bienaymé-Galton-Watson Branching Processes

Let *I_n_* be the incidence of clinical (or diagnosed) cases at time *n* and *Y_n,i_* represent the numbers of secondary cases generated at time *n* by the previous case *i*. Then *I_n_* is modelled from the past incidences by

(1) $$I_{n}=\sum_{i=1}^{I_{n-1}}Y_{n,i},$$

where the variables {*Y_n,i_*}*_i_* are assumed i.i.d. (independently and identically distributed) according to a distribution L independent of the time and of the past process given *F_n−_*_1_ := {*I_k_*}*_k≤n−_*_1_. We denote *m,* *σ*^2^ the mean and variance of *Y_n,_*_1_ given *F_n−_*_1_. Since these first two moments are the moments influencing the behavior of the process on the non-extinction set, we will also write $L(m,\sigma^{2})$ rather than L. This comes from the following writing of (1): $I_{n}=mI_{n-1}+\sqrt{I_{n-1}}\eta_{n}$, where $\eta_{n}\;:=[\sum_{i=1}^{I_{n-1}}(Y_{n,i}-m)]{\left[\sqrt{I_{n-1}}\right]}^{-1}$ is asymptotically distributed according to $N(0,\;\sigma^{2})$ on {lim*_n_* *I_n_* = ∞}, which is the non-extinction set, as *n* → *∞*.

The behavior of this process has been deeply analysed for a long time (see for example [1, 13, 16, 24]). We recall here the main properties. The trajectories of this process either die out or explode in an exponential way: *P*({lim*_n_* *I_n_* = *∞*} ∪ {lim*_n_* *I_n_* = 0}) = 1 and there exists an integrable random variable *W* such that lim*_n_* *I_n_m*^−*n*^ = *W*, a.s. (almost surely), where *P*(*W* > 0) > 0 if and only if *m* > 1 (supercritical case). The random variable *W* depends on *I*_0_ and of $L(m,\sigma^{2})$. This limit behavior comes from the martingale property of *I_n_m*^−*n*^, that is *E*(*I_n_m*^−*n*^*|F*_*n*−1_) = *I*_*n*−1_*m*^−(*n*−1)^ which implies *E*(*I_n_m*^−*n*^) = *I*_0_. So this process reproduces the initial phase of exponential growth of an epidemic and can be used for describing this phase when the incubation period is negligible compared to the time unit. The quantity *m* := *E*(*Y*_*n,*1_*|F*_*n*−1_) is the current reproductive number of the process (mean number of secondary cases produced by one case during a time unit) and is known to be the bifurcation parameter of the process, that is, *m* ≤ 1 implies the a.s. extinction of the process. The deterministic model *X_n_* = *mX*_*n*−1_ with *X*_0_ = *I*_0_, derived from *E*(*I_n_|F*_*n*−1_) = *I*_*n*−1_*m*, has the same bifurcation parameter, but when *m* = 1, the deterministic model persists with *X_n_* = *I*_0_ while the stochastic one dies out a.s.. Moreover before dying out, the stochastic process *I_n_|I_n_* ≠ 0 presents a linear increasing behavior: lim*_n_* *P*(*I_n_*[0.5*σ*^2^*n*]^−1^ ≤ *x|I_n_* ≠ 0) = 1 − exp(−*x*).

In addition, the probability of extinction, *q*, is the solution of *q* = *f*(*q*) := *E*(*q*^*Y*_1,1_^) and in the subcritical/critical cases *m* ≤ 1, the “epidemic size” $N\;:=\sum_{k=0}^{\text{Text}.}I_{k}$ (total number of cases generated until the extinction time *T_ext._*), follows a power series distribution when *Y*_*n,*1_ follows itself a power series distribution, that is, *P*(*Y_n,i_* = *k*) ∝ *a_k_λ^k^* [7, 11, 12]. In particular, when *P*(*Y_n,i_* = *k*) = exp(−*m*)[*k*!]^−1^*m^k^* (*Poisson*(*m*) distribution), then *N* follows the *Borel − Tanner*(*m, I*_0_) distribution, for *m* < 1,

(2) $$P(N=k)=\text{exp}(-mk)\frac{I_{0}}{k}\frac{{\left(mk\right)}^{k-I_{0}}}{(k-I_{0})!},\;\;\;k\geq I_{0},$$

and *E*(*N*) = *I*_0_(1 − *m*)^−1^, *Var*(*N*) = *I*_0_*m*(1 − *m*)^−3^ are increasing functions of *m*, *I*_0_.

## 3. Single-type Branching Processes with Population-Dependent Offsprings

When the population is small or when the individual migrations are slow compared to the infection process, the depletion of susceptible individuals due to the infection should be taken into account, which is not the case in the BGW process. The typical bell curve form of outbreaks is due to this depletion. The simplest such model is just an extension of the BGW process, that is, $I_{n}=\sum_{i=1}^{I_{n-1}}Y_{n,i}$, where the {*Y_n,i_*}*_i_* are assumed i.i.d. according to a distribution $L(F_{n-1})=:\;L(m(F_{n-1}),\;\sigma^{2}(F_{n-1}))$ given *F*_*n*−1_ : {*I_k_*}_*k*≤*n*−1_. The depletion effect of the *S* population at time *n* is replaced by the fact that *m*(*F*_*n*−1_) (resp. *P*(*Y_n,i_* = 0*|F*_*n*−1_)) is a decreasing (resp. increasing) function of the previous incidences of cases {*I_k_*}_*k*≤*n*−1_.

A simple example is when $L(F_{n-1})=L(\sum_{l=1}^{d_{n}}\mu^{l}I_{n-l})$, *μ* ∈ [0, 1], where $m(\sum_{l=1}^{d_{n}}\mu^{l}I_{n-l})\;(\text{resp}.\;P(Y_{n,1}=0|F_{n-1})=p(\sum_{l=1}^{d_{n}}\mu^{l}I_{n-l}))$ is a decreasing (resp. increasing) function of $\sum_{l=1}^{d_{n}}\mu^{l}I_{n-l}$. For example, *E*(*Y*_*n*,1_|*Y*_*n*,1_ ≠ 0) = *α* and $P(Y_{n,1}=0|F_{n-1})=1-K{\left[K+\sum_{l=1}^{d_{n}}\mu^{l}I_{n-l}\right]}^{-1}$, 0 < μ ≤ 1, *K* > 0. We will see in this section that this kind of models may exhibit a random cycle and therefore may be used for modeling recurrent epidemics. The extremal case *μ* = 0 means no depletion in *S*, which is got by an immediate healing with no immunization, after one time unit in the state *I* (*SIS* disease) and this model is reduced to the previous BGW process.

The other extremal case *μ* = 1 with *d_n_* = *n* corresponds either to a not necessarily immediate healing with persistent immunization or to a fatal issue (*SIR* disease). This process is a branching process with a long memory and has not be studied in the literature excepted by simulation [27].

The intermediate case 0 < *μ* ≤ 1 with *d_n_* = *d* represent the possibility of recovering but with a transient immunization only, and leads to a Markovian chain of order *d*. The behavior of this process has been studied in [27] and is described in the following paragraphs.

Let *A*1: there exists a positive function *f*(.) such that *P*(*Y_n,i_* = 0*|I*_*n*−1_ = *N, F*_*n*−1_) ≥ *f*(*N*) > 0, for all *N* ∈ ℕ_+_ and any value of *F*_*n*−1_ consistent with *I*_*n*−1_ = *N*.

This assumption is checked as soon as *P*(*Y_n,i_* = 0*|F*_*n*−1_) is a non-decreasing function of *I*_*n*−1_, *I*_*n*−2_,. . ., which is strictly increasing in *I*_*n*−1_.

**Proposition 1** *Let us assume A*1*. Then P*(lim*_n_* *I_n_* = 0 ∪ lim*_n_* *I_n_* = ∞) = 1.

Let *A*2: there exists m_*_ < ∞ such that *m*(*F*) ≤ m_*_, for all *F*, and lim_*|F|*→∞_*m*(*F*) < 1, where *|F|* is the *L*_1_-norm of *F*.

Let us define the bifurcation parameter *R_∞_* by

$$R_{\infty }=\text{sup}\{R:0<R<1\Rightarrow P(\underset{n}{\text{lim}}\;I_{n}=0)=1\},$$

where *R* is any real quantity depending on the parameters of the distribution of {*I_n_*}.

**Proposition 2** *The bifurcation parameter R*_∞_ *is equal to* lim_*|F|*→∞_*m*(*F*)*. Let us assume A*1 *and A*2*. Then R*_∞_ < 1*, i.e., the process dies out a.s.. Moreover for the deterministic trajectory* {*X_n_*} *defined by* $X_{n}\;:=m(F_{n-1}^{X})X_{n-1}$, *where* $F_{n-1}^{X}=\{X_{n-1},\;X_{n-2},\;\ldots \}$, *then the bifurcation parameter is the reproductive number R*_0_ = lim_*|F*^*X*^*|*→0_*m*(*F^X^*)*, that is, if R*_0_ < 1*, then* lim*_n_* *X_n_* = 0*, while if R*_0_ > 1*, then* lim*_n_X_n_* ≠ 0.

**Remark 1** *When d_n_* = *d, for all n,* {*I_n_*} *may be represented as a multitype branching process* **Ĩ***_n_* = (*I_n_, I_n−_*_1_*, ..., I_n−_*_(_*_d−_*_1)_) =: (*I_n,_*_1_*, I_n,_*_2_*, ..., I_n,d_*)*, which is asymptotically BGW if* $L(F_{n-1})$ *tends to a distribution* L *independent of the process as time tends to ∞ on* {lim*_n_* *I_n_* = ∞}*. But the asymptotic BGW process is not positively regular at the opposite of classical BGW branching processes, since* **M͂**(*F*_*n*−1_) *being defined by E*(**Ĩ***_n_|F*_*n*−1_) =: **Ĩ**_*n*−1_**M͂**(*F*_*n*−1_)*, then*

$$\tilde{\text{M}}(F_{n-1})=\left(\begin{array}{lllll} m(F_{n-1}) & 1 & 0 & \ldots & 0\\ 0 & 0 & 1 & \ldots & 0\\ \ldots & \ldots & & & \\ 0 & 0 & 0 & \ldots & 1\\ 0 & 0 & 0 & \ldots & 0 \end{array}\right)$$

*which implies that, for all n* ≥ *d* − 1, **M͂***^n^*[1*, j*] = *m*^*n*−(*j*−1)^*, and* **M͂***^n^*[*i, j*] = 0*, i* > 1*, where m* := lim_*|F|*→∞_ *m*(*F*) *and* **M͂** := lim_*|F|*→∞_ **M͂**(*F*).

**Remark 2** *If m*((1, 0, ..., 0)) > 1 *(supercritical assumption at the beginning of the disease spread), since m*(*F*_*n*−1_) < 1 *under A*2*, for |F*_*n*−1_*| and n sufficiently large, then we may observe oscillations until the a.s. extinction, the process increasing when |***Ĩ**_*n*−1_|*is small enough, and decreasing when it is too large. It is the case of the logistic model* $m(F_{n-1})=\alpha K{\left[K+\sum_{l=1}^{d_{n}}\mu^{l}I_{n-l}\right]}^{-1}$, *when d_n_* = *d with d* > 1 *and m*((1*,* 0*, ...,* 0)) = *α*(1 + *μK*^−1^)^−1^ > 1*. Moreover since R*_0_ = *α* > 1, *then* {*X_n_*} *tends to a stable limit cycle, as n* → ∞ [9, 27].

## 4. Set of Single-type Branching Processes with Population-Dependent Offsprings

We generalize here the model of the previous section to a set of *J* similar diseases in competition:

$$I_{n}^{\left(j\right)}=\sum_{i=1}^{I_{n-1}^{\left(j\right)}}Y_{n,i}^{\left(j\right)},\;\;j=1,\;\ldots ,\;J,$$

where, for each *j*, the ${\left\{Y_{n,i}^{\left(j\right)}\right\}}_{i}$ are i.i.d. according to $L^{\left(j\right)}(F_{n-1})=:L(m^{\left(j\right)}(F_{n-1}),\sigma^{2(j)}(F_{n-1}))$ given $F_{n-1}\;:\;{\left\{{\left\{I_{k}^{\left(j\right)}\right\}}_{j=1,\ldots J}\right\}}_{k\leq n-1}$. We assume that *m*(*F*_*n*−1_) is non increasing in $I_{k}^{\left(j\right)}$, for each *j*, each *k* ≤ *n* − 1.

A simple example is when as previously $L^{\left(j\right)}(F_{n-1})=L^{\left(j\right)}(\sum_{l=1}^{d_{n}}\mu^{l}\sum_{j^{\prime }}I_{n-l}^{\left(j^{\prime }\right)})$, *μ* ∈ [0, 1], and $m^{\left(j\right)}(\sum_{l=1}^{d_{n}}\mu^{l}\sum_{j^{\prime }}I_{n-l}^{\left(j^{\prime }\right)})\;(\text{resp}\text{.}\;P(Y_{n,1}^{\left(j\right)}=0|F_{n-1})=p^{\left(j\right)}(\sum_{l=1}^{d_{n}}\mu^{l}\sum_{j^{\prime }}I_{n-l}^{\left(j^{\prime }\right)}))$ is a decreasing (resp. increasing) function of $\sum_{l=1}^{d_{n}}\mu^{l}\sum_{j^{\prime }}I_{n-l}^{\left(j^{\prime }\right)}$. A typical example is given by different influenza viruses.

**Figure 1.**
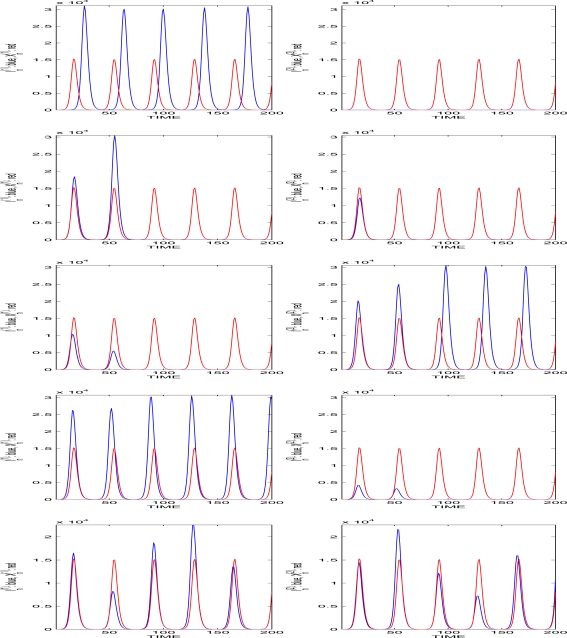
Two populations of infectives from similar diseases in competition following the same logistic Poisson model $I_{n}^{\left(j\right)}=\sum_{i=1}^{I_{n-1}^{\left(j\right)}}Y_{n,i}^{\left(j\right)}$, $Y_{n,i}^{\left(j\right)}|F_{n-1}∼\text{Poisson}(\alpha K{\left[K+\sum_{l\leq d}\mu^{l}(I_{n-l}^{\left(1\right)}+I_{n-l}^{\left(2\right)})\right]}^{-1})$, *j* = 1*,* 2, with $I_{0}^{\left(1\right)}=I_{0}^{\left(2\right)}=1$, *K* = 10^5^, *μ* = 1, *d* = 20. Each line of graphics concerns a trajectory of the process $\left\{I_{n}^{\left(1\right)},\;I_{n}^{\left(2\right)}\right\}$, and on each line, the graphic on the left concerns population 1 and the graphic on the right, population 2. On each graphic, the red line represents the deterministic limit cycle (reached very quickly) and the blue one, the stochastic cycle. We see that when one population is small during a long enough period, then the other population may be large, but both populations may also die out very quickly (second line).

As in the single-type case, assuming *m*^(*j*)^(*F*) < m_*_ < ∞, for all *F*, with $R_{\infty }^{\left(j\right)}\;:={\bar{\text{lim}}}_{|F|\to \infty }m^{\left(j\right)}(F)\;<\;1$, then, for all *j*, $P({\text{lim}}_{n}\;I_{n}^{\left(j\right)}=0)=1$, that is each disease dies out. Therefore as soon as the number of cases due to any pathogenic agent decreases or dies out, then the epidemics due to the other pathogenic agents may increase (see Figure 1).

Let the deterministic associate trajectory $\left\{X_{n}^{\left(j\right)}\right\}$ be defined by $X_{n}^{\left(j\right)}\;:=m^{\left(j\right)}(F_{n-1}^{X})X_{n-1}^{\left(j\right)}$, *n*≥ 1, $X_{0}^{\left(j\right)}\;:=I_{0}^{\left(j\right)}$.

**Proposition 3** *The reproductive number* $R_{\infty }^{\left(j\right)}\;:={\bar{\text{lim}}}_{|F|\to \infty }m^{\left(j\right)}(F)$ *is the bifurcation parameter for* $\left\{I_{n}^{\left(j\right)}\right\}$*, that is, if* $R_{\infty }^{\left(j\right)}<1$*, then* ${\text{lim}}_{n}I_{n}^{\left(j\right)}\overset{a.s.}{=}0$*, and the reproductive number* $R_{0}^{\left(j\right)}\;:={\underline{\text{lim}}}_{|F^{X}|\to 0}m^{\left(j\right)}(F^{X})$ *is the bifurcation parameter of* $\left\{X_{n}^{\left(j\right)}\right\}$*, that is if* $R_{0}^{\left(j\right)}<1$*, then* ${\text{lim}}_{n}X_{n}^{\left(j\right)}=0$*. Moreover if* $R_{0}^{\left(j\right)}\;>\;1$*, then* ${\bar{\text{lim}}}_{n}X_{n}^{\left(j\right)}\;>\;0$.

A possible extension of the previous models consists in adding an immigration. Then:

(3) $$I_{n}=\sum_{i=1}^{I_{n-1}}Y_{n,i}+J_{n}\delta_{n},$$

where *J_n_* is an immigration at time *n* when *δ_n_* = 1, and *δ_n_* is a Bernoulli variable allowing or not an immigration at time *n*. When {*δ_n_*} follows some seasonality, then the model is suitable for recurrent seasonal epidemics with seasonal immigrant, such as influenza.

This type of processes has been deeply studied in the subcritical case *m* < 1, when ({*Y_n,i_*}*_i_, J_n_*) has a distribution independent of *n* and *F*_*n*−1_, with either an immigration allowed only in the periods of extinction of the process {*I_n_*} (regenerative branching process), or when the {*δ_n_*} are i.i.d. independent of *F*_*n*−1_ (see the review article [39] and the estimation point of view in [25]).

We may also write (3) in the following way:

$$I_{n}=1_{\left\{I_{n-1}\neq 0\right\}}\sum_{i=1}^{I_{n-1}}{\tilde{Y}}_{n,i}+1_{\left\{I_{n-1}=0\right\}}J_{n}\delta_{n},\;\;\;{\tilde{Y}}_{n,i}=Y_{n,i}+\frac{J_{n}}{I_{n-1}}\delta_{n}.$$

Consequently, when ({*Y_n,i_*}*_i_, J_n_, δ_n_*) is not time-dependent and when 1_{*I*_*n*−1_=0}_*J_n_δ_n_* = 0, for all *n*, then (3) may be written as a model of Section 3..

## 5. Multitype Branching Processes with Age and Population-Dependent Offsprings

Let ${\text{N}}_{n}=(N_{n}^{1},\;\ldots N_{n}^{D})$ satisfying

(4) $$N_{n}^{k}=\sum_{h=1}^{D}\sum_{l=1}^{d}\sum_{i=1}^{N_{n-l}^{h}}Y_{n-l,n,i}^{(h),k},\;\;k=1,\;\ldots ,\;D,$$

where *h*, *k* represent types and *l* is the maturation time for getting the “offsprings” $Y_{n-l,n,i}^{(h),k}$ of the individual *i* (number of *k* individuals at time *n* generated by *i* belonging to the type *h* at time *n − l*). We assume that the ${\left\{Y_{n-l,n,i}^{(h),k}\right\}}_{i,l}$ are mutually independent given *F_n−1_* = {**N***_n−1_*, ..., **N**_0_} and that the ${\left\{Y_{n-l,n,i}^{(h),k}\right\}}_{i}$ are i.i.d. given *F_n−1_*.

The models of the previous sections (Sections 2. and 4.) belong to this class when *d_n_* = *d*.

When rigorously modeling the propagation of a disease in a given population, taking explicitly into account the population dynamic and the disease dynamic, then a type should be a health state (*S*, *E* (incubation) or *I*, for example) crossed with an influence factors level, and the “offsprings” $\left\{Y_{n-l,n,i}^{(h),k}\right\}$ should be expressed according to the number of newborn individuals of *i*, who are of the *k* type, and to the proper transition of *i* from the state *h* at *n − l* to the state *k* at *n*. For example assuming that there is no influence factors and that the set of individual health states is {*S, E, I*} and assuming that the life duration in the state *I* is at most one time unit, then, for *k* = *I*,

$$Y_{n-l,n,i}^{(h),I}=1_{\left\{h=S\right\}}\delta_{2,n-l+1,n,i}^{E,I|h}+\sum_{j=1}^{Y_{n-l+1,i}^{\left(h\right)}}\delta_{1,n-l+1,n,i,j}^{E,I|(h)},$$

where the ${\left\{\delta_{2,n-l+1,n,i}^{E,I|S}\right\}}_{i}$ are i.i.d. Bernoulli variables given *F_n−1_*, with $\delta_{2,n-l+1,n,i}^{E,I|S}$ equal to 1 if the individual *i* undergoes transitions *S* → *E*, at time *n − l* + 1, and *E* → *I*, at time *n*, which means that his incubation period is *l*, the ${\left\{\delta_{1,n-l+1,n,i,j}^{E,I|(h)}\right\}}_{i,j}$ are similar i.i.d. Bernoulli variables given *F_n−1_*, relative to the newborn *j* of *i*, and the ${\left\{Y_{n-l+1,i}^{\left(h\right)}\right\}}_{i}$ are i.i.d variables given *F_n_*−1, $Y_{n-l+1,i}^{\left(h\right)}$ being the number of newborns of *i* at time *n − l* + 1. So $Y_{n-l,n,i}^{(h),I}$ is the number of *I* individuals generated at time *n* with an incubation time *l* from *i* who is in the *h* type at *n − l*.

Let us write ℕ*_n_* := (**N***_n_*, **N***_n−1_*, ..., **N**_*n−(d−1)*_). Then *E*(ℕ*_n_|F_n−1_*) = ℕ*_n−1_* M(*F_n−1_*), where

$$\text{M}(F_{n-1})=\left(\begin{array}{lllll} {\text{M}}_{n-1,n}(F_{n-1}) & \text{I} & 0 & \ldots & 0\\ {\text{M}}_{n-2,n}(F_{n-1}) & 0 & \text{I} & \ldots & 0\\ \ldots & \ldots & & & \\ {\text{M}}_{n-(d-1),n}(F_{n-1}) & 0 & 0 & \ldots & \text{I}\\ {\text{M}}_{n-d,n}(F_{n-1}) & 0 & 0 & \ldots & 0 \end{array}\right)$$

**I** is the *D × D* identity matrix, and ${\text{M}}_{n-l,n}(F_{n-1})[h,\;k]\;:=E(Y_{n-l,n,i}^{(h),k}|F_{n-1})$, for *h* = 1, ..., *D*, *k* = 1, ..., *D*. Then, assuming that M(*F*) is invertible, for any value *F* of *F_n−1_*, ℕ*_n_*[ M(*F*_0_)... M(*F_n−1_*)]^−1^ is a martingale with *E*(ℕ*_n_* [ M(*F*_0_)... M(*F_n−_*_1_)]^−1^|*F*_0_) = ℕ_0_, implying by the convergence theorem for martingale [15] that lim*_n_* ℕ*_n_* [ M(*F*_0_)... M(*F_n−_*_1_)]^−1^ U*^t^* = *W*_U_, for any vector U, where *W*_U_ is an integrable random variable. But, except in the simple cases M(*F_n−_*_1_) = M (BGW process) or $\text{M}(F_{n-1})=\text{M}(\sum_{k}N_{n-1}^{k})$ with lim*_N_* M(*N*) = *M* (asymptotically BGW process) [30, 31, 32, 33], we cannot deduce from this martingale convergence any information on the asymptotic behavior of {**N***_n_*} itself.

In Section 5.1., we give some approaches for studying the behavior of model (4) when the total population size remains bounded. In Sections 5.2. and 5.3., we study the asymptotic behavior of limit models derived from (4) as the initial population size increases to infinity.

### 5.1. The Population Size Remains Bounded

Let $N_{n}\;:=\sum_{k}N_{n}^{k}$ be the total population size at time *n*. Let us first assume that this population size remains bounded by some control. For example the population is a herd of farm animals. We assume that the number of newborns *Y_n,i_* at time *n* of the animal *i* is bounded whatever *i*, *n*, and we use the following control: if *N_n−_*_1_ > *N_M_*, for some chosen threshold *N_M_*, then *Y_n,i_* = 0 (all the newborn animals are sold), and when *N_n−_*_1_ < *N_m_*, for some chosen threshold *N_m_* < *N_M_*, then new animals are bought. Therefore assuming that *P*(ℕ*_n_* = *N*_2_*|*ℕ*_n−1_* = *N*_1_) =: *Q*(*N*_1_*, N*_2_) is independent of *n*, {ℕ*_n_*} is a homogeneous Markov chain on a finite space. Since the population is open to immigration, then the healthy state “0 case” is not an absorbing state and there may exist some endemic behavior: if there exists an asymptotic distribution *π*, it satisfies

$$\sum_{N_{1}}Q(N_{1},N_{2})\pi (N_{1})=\pi (N_{2}),\;\forall N_{2}\in {\text{N}}^{dD}.$$

But due to combinatorial aspects, the transition probabilities {*Q*(*N*_1_*, N*_2_)} may be difficult to compute. A solution is then to work in continuous time: in [26], we determine the distribution of the population process from the individual transitions that may be age and population-dependent and that may be all different, and in [40], we use this type of process for studying the propagation of the BVD (Bovine Diarrhoea Virus) within a dairy herd. For that, a renewal approach is used, which generalizes to a population the semi-Markov process theory relative to one individual. This approach also allows to build a rigorous and general simulation algorithm for this individual based model, but when used for non bounded branching populations, it cannot lead to fine stochastic behaviors such as ${\text{lim}}_{n}N_{n}{\left[N_{0}\rho^{n}\right]}^{-1}\overset{a.s.}{=}\;W$ determined by martingale theory in the frame of BGW processes.

### 5.2. The Initial Population Size N_0_ is Large and the Disease is not Rare

We assume here that the initial population size *N*_0_ is large which allows to study the limit, as *N*_0_ → ∞, of the following quantities: **N***_n_/N*_0_ =: **D̂***_n_* (empirical densities) or **N***_n_/N_n_* =: **P̂***_n_* (empirical probabilities) or **N***_n_*[*N*_0_*ρ^n^*]^−1^ =: **W***_n_* (empirical normalized densities), when assuming that lim_*N*_0__ **D̂**_0_ = **D**_0_ or lim_*N*_0__ **P̂**_0_ = **P**_0_.

A simple theoretical example that we (artificially) apply here on epidemics is the following model on densities with *D* = 1, *d* = 1 [34, 35, 36, 37, 38]: $I_{n}=\sum_{i=1}^{I_{n-1}}Y_{n,i}$, where the {*Y_n,i_*}*_i_* are i.i.d. given *F_n−1_* = {*I_n−1_*, *..., I*_0_} and depend on the previous cases incidences only through the condition: *Y_n,i_* = 0 when *I_n−1_* > *cI*_0_, that is, a massive vaccination is done as soon as the density $I_{n-1}I_{0}^{-1}$ crosses the threshold *c*. This implies that the process dies out a.s.. The author studied *D̂_n_* := *I_n_*/*I*_0_ and proved that lim_*I*_0__ lim*_n_* *D̂_n_*/*D̂_n_* ≠ 0 belongs to the set of stable fixed points of *D_n_*, where *D_n_* is the deterministic limit model *D_n_* = *D_n−_*_1_*m*(*D_n−_*_1_), *n* ≥ 1, *D*_0_ = 1, with *m*(*D_n−_*_1_) := *E*(*Y_n,_*_1_*|D̂_n−_*_1_ = *D_n−_*_1_). Thus, for *I*_0_ very large (which occurs when the initial time is chosen when the epidemic is large enough), the random empirical densities has the same asymptotic behaviour, as *n* → ∞, as the limit densities.

Let us study now the empirical probabilities in the general case *D* ≥ 1, *d* ≥ 1 under a density-dependence assumption. We prove in Proposition 5 that ${\text{lim}}_{n}{\text{lim}}_{N_{0}}{\hat{\text{P}}}_{n}|N_{n}\neq 0\overset{P}{=}{\text{lim}}_{N_{0}}{\text{lim}}_{n}{\hat{\text{P}}}_{n}|N_{n}\neq 0$ (and similarly for {**W***_n_*}), which allows to approximate lim*_n_* **P̂***_n_* or lim*_n_* **W***_n_*.

Let us first study the behavior of the total population size, under the assumption that the number of newborns is independent of the mother health state.

**Proposition 4** *Let us assume that the distribution of* $\sum_{k=1}^{D}Y_{n-l,n,1}^{(h),k}$ *only depends on l and is denoted* $\sum_{k=1}^{D}Y_{n-l,n,i}^{(h),k}=Y_{n-l,n,i}$*. Let us denote* $\Phi_{l}\;:=E(Y_{n-l,n,1}|F_{n-1}),\;\sigma_{l}^{2}\;:=Var(Y_{n-l,n,1}|F_{n-1})$*. Then the total size of the alive population at time n,* $N_{n}=\sum_{k=1}^{D}N_{n}^{k}$*, may be expressed as a multitype branching process* **Ñ***_n_* := (*N_n,_*_1_*, ...N_n,d_*) := (*N_n_, N_n−_*_1_*, ..., N_n−_*_(_*_d−_*_1)_)*, where* $N_{n}=\sum_{l=1}^{d}\sum_{i=1}^{N_{n-l}}Y_{n-l,n,i}$ *and*

$$N_{n,j}=\sum_{l=1}^{d}\sum_{i=1}^{N_{n-1,l}}Y_{n,i}^{\left(l,j\right)},$$

*with, for j* = 1, $Y_{n,i}^{\left(l,1\right)}=Y_{n-l,n,i}$, *for j* > 1, $Y_{n,i}^{\left(j-1,j\right)}=1$ *and* $Y_{n,i}^{\left(l,j\right)}=0$, *for l* ≠ *j* − 1, *and the* ${\left\{Y_{n,i}^{\left(l,j\right)}\right\}}_{i}$ *are independent given F_n−1_*.

*Moreover, assuming* 0 < Φ_*l*_ < ∞, *l* = 1, …, *d*, $0<\sum_{l=1}^{d}\sigma_{l}^{2}<\infty$*, and P*(*Y_n−l,n,i_* ≥ 2*|F_n−_*_1_) > 0 *for some l, then* {*N_n_*}*_n_* *satisfies the following properties:*

1. {*N_n_*} *is positively regular, nonsingular, and checks the xlogx property;*
2. *P*(lim*_n_* *N_n_* = 0 ∪ lim*_n_* *N_n_* = ∞) = 1
3. *Let ρ be the* *first eigenvalue of* **M͂** *defined by E*(**Ñ***_n_*|**Ñ***_n_*_−1_) =: **Ñ***_n_*_−1_**M͂**, *that is,*
  $$\tilde{\text{M}}=\left(\begin{array}{lllll} \Phi_{1} & 1 & 0 & \ldots & 0\\ \Phi_{2} & 0 & 1 & \ldots & 0\\ \ldots & \ldots & & & \\ \Phi_{d-1} & 0 & 0 & \ldots & 1\\ \Phi_{d} & 0 & 0 & \ldots & 0 \end{array}\right)$$
  *Then E*(**Ñ***_n_****ξ****^t^*) = **Ñ**_0_*ρ^n^****ξ****^t^*, *where* **M͂*ξ****^t^* = *ρ****ξ****^t^. Moreover ρ* ≤ 1 *(subcritical and critical cases) implies that P*(lim*_n_* *N_n_* = 0) = 1 *(a.s. extinction), and ρ* > 1 *(supercritical case) implies the existence of an integrable random variable W such that* ${\text{lim}}_{n\to \infty }W_{n}\overset{a.s.}{=}W$ *, where W_n_* := *N_n_*[*N*_0_*ρ^n^*]^−1^*, and P*(lim*_n_* *N_n_* = ∞) = *P*(*W* > 0)*. Finally let us assume that N*_−_*_l_* = *ρ*^−l^*N*_0_, *l* = 1, ..., *d* − 1. *Then if ρ* ∈ Q, ${\text{lim}}_{N_{0}\to \infty }W_{n}\overset{a.s.}{=}{\text{lim}}_{N_{0}\to \infty }{\text{lim}}_{n\to \infty }W_{n}\overset{a.s.}{=}1$.
4. *ρ is solution of* $\sum_{l=1}^{d}\Phi_{l\rho^{-l}}=1$*, and ρ* ≤ 1 ⇔*R*_∞_ ≤ 1*, where* $R_{\infty }=\sum_{l=1}^{d}\Phi_{l}$ *(total mean number of offspring generated by an individual).*

Let us notice that the process {*N_n_*} is the counterpart in discrete time of a single-type age-dependent Crump-Mode-Jagers branching process in continuous-time [29].

Let us write *α_l_* := Φ*_l_ρ^−l^*, *l* = 1*, ..., d*, *d_n_* := *d* ∧ *n*, *n_d_* = ⌊*n/d*⌋ (integer part of *n/d*) and let us define:

*A*3: ${\text{lim}}_{n}\rho^{-n/2}\sum_{k=n_{d}+1}^{n}\sum_{l_{1}\;,\ldots ,l_{k}}[\alpha_{l_{1}}\rho^{l_{1}/2}\ldots \alpha_{l_{k}}\rho^{l_{k}/2}]1_{\left\{\sum_{j=1}^{k}l_{j}\leq n\right\}}<\infty$.

For *d* = 1, or *d* > 1 with *φ_l_* = 0, for all *l < d*, then the sum in *A*3 is reduced to 0 implying that *A*3 is satisfied. In the general case *A*3 is satisfied under the stronger assumption:

$$\sum_{k=n_{d}+1}^{n}\sum_{l_{1}\;,\ldots ,l_{k}}[\alpha_{l_{1}}\ldots \alpha_{l_{k}}]1_{\left\{\sum_{j=1}^{k}l_{j}\leq n\right\}}<\infty ,$$

where, we notice that, for all *k* ≤ *n*, $\alpha_{1}^{k}\leq \sum_{l_{1}\;,\ldots ,l_{k}}\left[\alpha_{l_{1}}\ldots \alpha_{l_{k}}\right]1_{\left\{\sum_{j=1}^{k}l_{j}\leq n\right\}}\leq 1$ and $\sum_{l_{1}\;,\ldots ,l_{k}}\left[\alpha_{l_{1}}\ldots \alpha_{l_{k}}\right]1_{\left\{\sum_{j=1}^{k}l_{j}\leq n\right\}}$ is decreasing in *k*.

**Proposition 5** *Let us assume, as in Proposition 4, that the distribution of* $\sum_{k=1}^{D}Y_{n-l,n,1}^{(h),k}$ *depends only on l. Let us moreover assume ρ* > 1, *A*3, **M***_n−l,n_*(*F_n−1_*) = **M***_n−l,n_*(**P̂***_n−_*_1_) *(density-dependence),* lim_*N*_0→∞__**P̂**_0_ = **P**_0_*, and N*_−*l*_ = *c_l_N*_0_*, l* = 1*, ..., d* − 1*, where c_l_* *is independent of N*_0_*. Then, for all η* > 0*,* lim_*N*_0__ *P*(*|***P̂***_n_* − **P***_n_|* > *η|N_n_* ≠ 0)=0 *and*

$$\begin{array}{l} \underset{N_{0}}{\text{lim}}\underset{n}{\text{lim}}P(|{\hat{\text{P}}}_{n}-{\text{P}}_{n}|>\eta |N_{n}\neq 0)=\underset{n}{\text{lim}}\underset{N_{0}}{\text{lim}}P(|{\hat{\text{P}}}_{n}-{\text{P}}_{n}|>\eta |N_{n}\neq 0)=0,\\ \underset{N_{0}}{\text{lim}}\underset{n}{\text{lim}}P(|{\text{W}}_{n}-{\text{P}}_{n}|>\eta |N_{n}\neq 0)=\underset{n}{\text{lim}}\underset{N_{0}}{\text{lim}}P(|{\text{W}}_{n}-{\text{P}}_{n}|>\eta |N_{n}\neq 0)=0, \end{array}$$

*where* **P***_n_* *is the vector of probabilities defined by:*

$${\text{P}}_{n}\;:=(\sum_{l}\sum_{h}P_{n-l}^{h}\rho^{-l}{\text{M}}_{n-l,n}(F_{n-1})[h,\;1],\;\ldots ,\sum_{l}\sum_{h}P_{n-l}^{h}\rho^{-l}{\text{M}}_{n-l,n}(F_{n-1})[h,D]),\;n\geq 1.$$

Proposition 5 allows to use the attractors of the dynamical model {**P***_n_*} as an approximation of the asymptotic behavior of the empirical frequencies if the initial population size is very large, and moreover generalizes the result of the BGW process: ${\text{lim}}_{N_{0}}{\text{lim}}_{n}N_{n}{\left[N_{0}\rho^{n}\right]}^{-1}\overset{a.s.}{=}1$ deduced from ${\text{lim}}_{n}N_{n}{\left[N_{0}\rho^{n}\right]}^{-1}\overset{a.s.}{=}W$ (Section 2.). But the main drawbacks of this approach consist in the loss of the population variability, since the quantities are normalized by *N*_0_ with *N*_0_ tending to infinity, and the loss of the possibility for the extinction time of any type to be finite. Moreover the global asymptotic stability of the healthy state for {**P***_n_*} (extinction of the disease propagation starting from any initial infected population size) may be difficult to prove, the difficulty increasing with the number *d* × *D* of dimensions. This global asymptotic stability was studied for the propagation of a *SIS* disease in a branching population (*D* = 2) [21] and for the one of a *SEI* disease (*D* = 3) [22]. Both studies assumed *d* = 1 and we showed that the bifurcation parameter for the disease process was based on the comparison of the capacity of infection and the capacity for the population to renew its susceptible population.

### 5.3. The Initial Population Size is Large and the Disease is Rare

Another approach for studying the asymptotic behavior of the process {**N***_n_*} is to study the asymptotic behavior of the limit model assuming now that the disease is rare at the initial time, which forbids the use of densities or probabilities as in Section 5.2. We present here such an approach generalizing the case of the BSE propagation at the scale of a country [28].

Let us recall model (4): $N_{n}^{k}=\sum_{l=1}^{d}\sum_{h=1}^{D}\sum_{i=1}^{N_{n-l}^{h}}Y_{n-l,n,i}^{(h),k}$, where the ${\left\{Y_{n-l,n,i}^{(h),k}\right\}}_{i,l}$ are mutually independent given *F_n−_*_1_ = {**N***_n−_*_1_*, ...,* **N**_0_} and the ${\left\{Y_{n-l,n,i}^{(h),k}\right\}}_{i}$ are i.i.d. given *F_n−_*_1_. We assume here that *h* and *k* are health states *H* and *K* crossed with age *a* of the individual. For *k* = *I* × *a*, where *I* represents here the first time unit in the clinical state, then the number of new clinical cases aged *a* at time *n* generated by an individual *i* with a delay of *l* time units, is thus defined:

$$Y_{n-l,n,i}^{(h),I\times a}:=1_{\left\{a>l\right\}}1_{\left\{h=S\times a-l\right\}}\delta_{2,n-l+1,n,i}^{E,I|h}+1_{\left\{a=l\right\}}\sum_{j=1}^{Y_{n-l+1,i}^{\left(h\right)}}\delta_{1,n-l+1,n,i,j}^{E,I|(h)},$$

where we assume that the ${\left\{\delta_{2,n-l+1,n,i}^{E,I|h}\right\}}_{i}$ are i.i.d. Bernoulli variables given *F_n−1_*, the ${\left\{\delta_{1,n-l+1,n,i,j}^{E,I|(h)}\right\}}_{i,j}$ are i.i.d. Bernoulli variables given *F_n−1_*, the ${\left\{Y_{n-l+1,i}^{\left(h\right)}\right\}}_{i}$ are i.i.d. given *F_n−_*_1_, and the ${\left\{\delta_{2,n-l+1,n,i}^{E,I|h}\right\}}_{i}$ and the ${\left\{\delta_{1,n-l+1,n,i,j}^{E,I|(h)}\right\}}_{i,j}$ are mutually independent given *F_n−1_*. Therefore the incidence of cases aged *a* at time *n* is given by:

(5) $$N_{n}^{I\times a}=\sum_{l}\left[1_{\left\{a>l\right\}}\sum_{h}\sum_{i=1}^{N_{n-l}^{h}}[1_{\left\{h=S\times a-l\right\}}\delta_{2,n-l+1,n,i}^{E,I|h}+1_{\left\{a=l\right\}}\sum_{h}\sum_{i=1}^{N_{Y,n-l+1}^{\left(h\right)}}\delta_{1,n-l+1,n,i,j}^{E,I|(h)}\right],$$

where $N_{Y,k}^{\left(h\right)}\;:=\sum_{i=1}^{N_{k-1}^{h}}Y_{k,i}^{\left(h\right)}$.

Moreover, if a *I* individual may survive in this state a longer time than a time unit, the infection process will depend on the total number of infectives including all the *I* individuals. In this case, let *k* = *I^tot^* × *a*, where *I^tot^* represents the clinical state (new or not), then

(6) $$N_{n}^{I^{tot}\times a}=\sum_{l}\sum_{i=1}^{N_{n-l}^{I\times a-l}}\delta_{n-l,n,i}^{I,I^{\text{tot}}|I\times a-l}+N_{n}^{I\times a}$$

where $\delta_{n-l,n,i}^{I,I^{tot}|I\times a-l}$ is a Bernoulli variable, equal to 1 if *i* survives in the state *I* from age *a − l* at *n − l* to age *a* at *n* at least. Since the distribution of ${\left\{N_{n}^{I^{tot}\times a}\right\}}_{a,n}$ is easily calculated from the distribution of ${\left\{N_{n}^{I\times a}\right\}}_{a,n}$ and since clinical cases are generally rapidly isolated and then removed from the infection process, and since moreover only the new cases are counted by surveillance systems, we will study here the limit of $N_{n}^{I\times a}$ given $F_{n-1}^{*}\;:=\{{\left\{N_{k}^{I}\right\}}_{k\leq n-1},{\left\{N_{k}\right\}}_{k\leq n-1},{\left\{N_{Y,k}\right\}}_{k\leq n}\}$, where $N_{Y,k}=\sum_{h}N_{Y,k}^{\left(h\right)}$. Of course, expressions similar to (5) and (6) can be written for $N_{n}^{E\times a}$ and $N_{n}^{E^{\text{tot}}\times a}$.

Let *δ_n−l,h_*(*i*) be the Bernoulli variable equal to 1 if *i* belongs to the *h* population at time *n − l*.

**Proposition 6** *Let us assume that* ${\text{lim}}_{N_{0}}\;N_{n}^{H\times a}$ *exist, for H ∈* {*I, E, I^tot^, E^tot^*} *and all a, and let us assume the following conditions, for l* ≥ 2,

$$\begin{array}{l} E(\delta_{n-l,S\times a-l}(i)\delta_{2,n-l+1,n,i}^{E,I|S\times a-l}|F_{n-1}^{*})=E(\delta_{n-l,S\times a-l}(i)\delta_{2,n-l+1,n,i}^{E,I|S\times a-l}|F_{n-l}^{*})=:\;p_{2,a,l|n-l}(F_{n-l}^{*})\\ \sum_{h}E(\delta_{n-l,h}(i)\delta_{1,n-l+1,n,i,j}^{E,I|(h)}|F_{n-1}^{*})=\sum_{h}E(\delta_{n-l,h}(i)\delta_{1,n-l+1,n,i,j}^{E,I|(h)}|F_{n-l}^{*})=:p_{1,a,l|n-l}(F_{n-l}^{*})\\ \underset{N_{0}}{\text{lim}}N_{n-l}p_{2,a,l|n-l}(F_{n-l}^{*})=\Psi_{2,a,l|n-l}N_{n-l}^{I},\;\;\underset{N_{0}}{\text{lim}}N_{Y,n-l+1}p_{1,a,l|n-l}(F_{n-l}^{*})=\Psi_{1,a,l|n-l}N_{n-l}^{I}, \end{array}$$

*where* Ψ_1,*a,l|n−l*_ *and* Ψ_2,*a,l|n−l*_ *are independent of* $F_{n-l}^{*}$. Then the limit process $I_{a,n}\;:\overset{D}{=}{\text{lim}}_{N_{0}}N_{n}^{I\times a}$ *of incidence of cases aged a at time n is a single-type Markovian process of order d with a Poissonian transition law:*

(7) $$I_{a,n}|I_{n-1},\;I_{n-2},\;\ldots \;\;\;\;∼\;\;\;\;\text{Poisson}(\sum_{l=1}^{d}\Psi_{a,l|n-l}I_{n-l}),$$

(8) $$\Psi_{a,l|n-l}\;\;\;\;:=\;\;\;1_{\left\{a>l\right\}}\Psi_{2,a,l|n-l}+1_{\left\{a=l\right\}}\Psi_{1,a,l|n-l},$$

*where I_n_* := ∑*_a_* *I_a,n_. Moreover we may write* $I_{n}=\sum_{l=1}^{d}\sum_{i=1}^{I_{n-l}}Y_{n-l,n,i}$*, where the* {*Y_n−l,n,i_*}*_i_* *are i.i.d. given F*_*n*−1_ = {*I_n−_*_1_*, I_n−_*_2_*, ...*} *with Y_n−l,n,_*_1_*|F_n−_*_1_ ∼ *Poisson*(Ψ*_l|n−l_*)*,* Ψ*_l|n−l_* = ∑*_a_* Ψ*_a,l|n−l_, and the* {*Y_n−l,n,i_*}*_i,l_* *are independent.*

As an example of such an approach, we studied the propagation of the BSE in Great-Britain by a general model of type (4). Since the time unit is large (one year), we used Proposition 6 with, for *l* ≥ 2, ${\tilde{F}}_{n-l}^{*}\;:=\{{\left\{N_{k}^{I}\right\}}_{k\leq n-l+1,}{\left\{N_{k}\right\}}_{k\leq n-l,}\;{\left\{N_{Y,k}\right\}}_{k\leq n-l+1}\}$ instead of $F_{n-l}^{*}$. Then the assumptions of this proposition 6 were satisfied under the following assumptions [28]:

1. The *S* and *E* individuals have the same time-homogeneous survival law {*S_a_*}*_a_*;
2. There is no over-contamination during the incubation period or the clinical state;
3. The number of newborn animals $Y_{l,i}^{\left(h\right)}$ at each birth per individual at time *l*, is independent of *l*, *i*, and of the health state *h* of *i* (but the health state of each newborn and his survival during the first time unit may depend on *h*);
4. The population is roughly stable: ${\text{lim}}_{N_{0}}N_{1}N_{0}^{-1}\overset{a.s.}{=}1$;
5. The disease is rare at the initial time: ${\text{lim}}_{N_{0}}{\left(N_{0}^{E}+N_{0}^{I}\right)}^{2}N_{0}^{-1}\overset{a.s.}{=}0$;
6. The probability for a given *S* to be infected at time *k* + 1 via the horizontal route of excretion, follows a Reed-Frost’s type model, that is
  $$\begin{array}{l} P(i\;\text{aged}\;a\;\text{at}\;k,\;\text{is}\;\text{in}\;\text{fected}\;\text{at}\;k+1\;\text{by}\;\text{excretion}|F_{k}^{*},\;i\;\text{survives}\;\text{at}\;k+1)=\\ E(1-{\left(1-\frac{\theta^{a,\;R^{c}}}{N_{k}}\right)}^{N_{k}^{I_{*}^{\text{tot}}}}|F_{k}^{*})\overset{N_{k}\;\text{large}}{≃}\;\theta^{a,\;R^{c}}\frac{E(N_{k}^{I_{*}^{\text{tot}}}|F_{k}^{*})}{N_{k}}, \end{array}$$
  where $N_{k}^{I_{*}^{\text{tot}}}$ is the number of infectious animals at time *k* (including those in the latest stage of their incubation period). We assume a similar expression for the infection via the horizontal route concerning contaminated meat and bone meal produced from dead infectious animals.

Under these assumptions, then $\Psi_{l|n-l}=\sum_{a=l}^{d}\left[(\theta^{a-l,R^{c}}+\theta^{a-l,R}\varphi_{n-l})+1_{\left\{a=l\right\}}p_{mat}\right]P(a)P_{inc.}(l)$, where $\theta_{l}^{a-l,R^{c}}$ and $\theta_{l}^{a-l,R}{\left(\text{exp}\;\lambda -1\right)}^{-1}\varphi_{n-l}$ represent the mean numbers of new infected animals aged *a − l* + 1 per infective at time *n − l* + 1 respectively via a horizontal route (excretion of alive animals and slaughtered animals respectively), *φ_n−l_* ∈ [0, 1] represents the efficiency at time *n − l* of the main current control regulations: *φ_k_* = 1, for *k* ≤ 1988, *φ_k_* = *φ*, for *k* ∈ (1989, 1996), and *φ_k_* = 0, for *k* ≥ 1997, where *φ* represents the efficiency of the Meat and Bone Meal ban of July 1988, *p_mat._* is the probability for a newborn animal to be infected by his mother (maternal route), *P*(*a*) is the probability at each time for an animal to have age *a* which may be expressed as a function of the survival distribution, and *P_inc._*(*l*) is the probability for an infected animal to have an intrinsic incubation time equal to *l* (conditioned on survival). So Ψ*_l|n−l_* represents the mean number of new clinical cases produced with a delay *l* by a clinical case of time *n − l*.

Let us assume that Φ*_l|n−l_* depends only on *l*. This is achieved by modeling the process on a period with a constant control. Then {*I_n_*} may be written as a multitype BGW branching process and therefore results of Proposition 4 are valid for this process, replacing Φ*_l_* by Ψ*_l_* := Ψ*_l|n−l_*. More precisely, let **Ĩ***_n_* =: (*I_n,_*_1_*, I_n,_*_2_*, ..., I_n,d_*) := (*I_n_, I_n−_*_1_*, ..., I_n−_*_(_*_d−_*_1)_) and *F_n−_*_1_ = {*I_n−_*_1_*, I_n−_*_2_*, ..., I*_0_}.

Let **M͂** be defined by *E*(**Ĩ***_n_|F_n−_*_1_) =: **Ĩ***_n−_*_1_**M͂**, where

$$\tilde{\text{M}}=\left(\begin{array}{lllll} \Psi_{1} & 1 & 0 & \ldots & 0\\ \Psi_{2} & 0 & 1 & \ldots & 0\\ \ldots & \ldots & & & \\ \Psi_{d-1} & 0 & 0 & \ldots & 1\\ \Psi_{d} & 0 & 0 & \ldots & 0 \end{array}\right)$$

**Proposition 7** {**Ĩ***_n_*_}_ *is a multitype BGW branching process:*

$$I_{n,j}=\sum_{l=1}^{d}\sum_{i=1}^{I_{n-1,l}}Y_{n,i}^{\left(l,j\right)},$$

*where, for j* = 1, $Y_{n,i}^{\left(l,1\right)}\;:=Y_{n-l,n,i}$, *for j* > 1, $Y_{n,i}^{\left(j-1,j\right)}:=1$ *and* $Y_{n,i}^{\left(l,j\right)}\;:=0$, *for l* ≠ *j* − 1*, and the* ${\left\{Y_{n,i}^{\left(l,j\right)}\right\}}_{i,l,j}$ *are independent given F_n_*_–1_ *and* $Y_{n,i}^{\left(l,1\right)}|{\tilde{\text{I}}}_{n-1}∼\text{Poisson}(\Psi_{l})$.

*Moreover E*(**Ĩ***_n_****ξ****^t^*) = **Ĩ**_0_*ρ^n^****ξ****^t^, where ρ and* ***ξ*** *are the first eigenvalue and corresponding eigenvector with ξ*_1_ = 1*, of* **M͂***, that is,* **M͂*ξ****^t^* = *ρ****ξ****^t^, implying that* $\xi_{l}=\rho^{l-1}\sum_{l^{\prime }=l}^{d}\rho^{-l^{\prime }}\Psi_{l^{\prime }}$, *l* = 1, …, *d*.

Let s := (*s*_1_, .., *s_d_*), *s_d_*_+1_ := 1, $f^{\left(l\right)}(\text{s})\;:=E(s_{1}^{Y_{n,\;1}^{\left(l,\;1\right)}}\ldots s_{d}^{Y_{n,\;1}^{\left(l,d\right)}})$, **f**(**s**) := (*f*^(1)^(**s**)*, ..., f*^(^*^d^*^)^(**s**)).

**Proposition 8** *The generating function of* {**Ĩ**_*n*_}, $F_{n}(s)\;:=E(s_{1}^{I_{n,1}}\ldots s_{d}^{I_{n,d}})$ *is equal to*

$$F_{n}(\text{s})=F_{n-1}(\text{f}(\text{s}))=F_{n-2}(\text{f}(\text{f}(\text{s})))=\ldots =F_{0}({\text{f}}_{n}(\text{s}))\;:={\left[f_{n,1}(\text{s})\right]}^{I_{0,1}}\ldots {\left[f_{n,d}(\text{s})\right]}^{I_{0,d}},$$

*where* **f***_n_*(**s**) := **f**(**f***_n−_*_1_(**s**))*, and f*^(^*^h^*^)^(**s**) = *s_h_*_+1_ exp(−Ψ*_h_*(1 *− s*_1_)).

As in Proposition 4, the bifurcation parameter of the process is given by the first eigenvalue *ρ* of **M͂**.

**Proposition 9** *ρ is solution of* $\sum_{l=1}^{d}\Psi_{l}\rho^{-1}=1$*, and ρ* ≤ 1 *is equivalent to R_∞_* ≤ 1*, where* $R_{\infty }=\sum_{l=1}^{d}\Psi_{l}$ *is the total mean number of new cases I who begin to be generated by a I during his first time unit.*

Let us assume from now on that Ψ_1_ > 0,..., Ψ*_d_* > 0.

**Proposition 10**

1. *P*(lim*_n_* *I_n_* = 0 ∪ lim*_n_* *I_n_* = ∞) = 1;
2. *ρ* > 1 *is equivalent to R*_∞_ > 1 *(supercritical case) which itself implies the existence of a positive integrable random variable W such that* ${\text{lim}}_{n\to \infty }I_{n}{\left[I_{0}\rho^{n}\right]}^{-1}\overset{a.s.}{=}W$, *where P*(lim*_n_* *I_n_* = ∞) = *P*(*W* > 0)*. Moreover let us assume that I*_−_*_l_* = *c_l_I_0_*, *l* = 1, ..., *d* − 1, where *c_l_* is independent of *I*_0_. Then ${\text{lim}}_{I_{0}\to \infty }I_{n}{\left[I_{0}\rho^{n}\right]}^{-1}\overset{a.s.}{=}{\text{lim}}_{I_{0}\to \infty }{\text{lim}}_{n\to \infty }I_{n}{\left[I_{0}\rho^{n}\right]}^{-1}\overset{a.s.}{=}1$.
3. *ρ* ≤ 1 *is equivalent to R*_∞_ ≤ 1 *(subcritical and critical cases) which implies P*(lim*_n_* *I_n_* = 0) = 1 *(a.s. extinction).*

Let us point out that the deterministic model derived from *E*(**Ĩ***_n_|***Ĩ***_n−_*_1)_ = **Ĩ***_n−_*_1_ **M͂** is defined by: **X͂***_n_* := **X͂***_n−_*_1_**M͂**, **X͂**_0_ := **Ĩ**_0._ Therefore **X͂***_n_* = **X͂**_0_**M͂***^n^* and the bifurcation parameter of {**X͂***_n_*} is *ρ*, but for *ρ* = 1, **X͂***_n_****ξ****^t^* = **X͂**_0_***ξ****^t^* (persistence), while ${\text{lim}}_{n}{\tilde{\text{I}}}_{n}\overset{a.s.}{=}0$ (extinction).

Let *T_ext._* be the (random) extinction time of {*I_n_*}. Then the extinction probability is *q* := *P*_**Ĩ**_0__(lim*_n_* **Ĩ***_n_* = 0) = *P*_**Ĩ**_0__(*T_ext._* *< ∞*).

**Proposition 11** *We have* $q=q_{1}^{I_{0}}\ldots q_{d}^{I_{-(d-1)}}$*, where* $q_{h}=\text{exp}(-\sum_{l=h}^{d}\Psi_{l}(1-q_{1}))$*, is the extinction probability starting from* **Ĩ**_0_ = (0*, ..,* 0*,* 1*,* 0*, ...,* 0) *(one individual of the h type), h* = 1*, ..., d.*

As a consequence, in the particular case **Ĩ**_0_ = (*I*_0_*,* 0*, ...,* 0), then $q=q_{1}^{I_{0}}$, where *q*_1_ is solution of the equation: 1 *− q*_1_ = 1 − exp(*−R_∞_*(1 *− q*_1_)). Thus, there exists a solution *q*_1_ ≠ 1 if *R_∞_* > 1, implying that *q*_1_ decreases as *R_∞_* increases. Otherwise, if *R_∞_* ≤ 1, then *q*_1_ = 1, implying *q_h_* = 1, for all *h* = 1*, ..., d*.

Let *G_n_* := *P*(*T_ext._* ≤ *n*) = *P*(**Ĩ***_n_* = **0**) (distribution of the extinction time *T_ext_*.). Then according to [1], p.187, if *R_∞_* *<* 1, the distribution of the extinction time has an exponential form as *n* → ∞: lim*_n_* *ρ*^−^*^n^*(1 *− G_n_*) = *Q*(**0**)**Ĩ**_0_.***ξ****^t^* > 0 where *Q*(**0**) := lim*_n_* *ρ*^−^*^n^****ζ***.(**1** − **f***_n_*(**0**))*^t^*, **M*ξ****^t^* = *ρ****ξ****^t^*, ***ζ*M** = *ρ****ζ***, ***ξ.ζ****^t^* = 1, ***ξ***.**1***^t^* = 1, and for *h* = 1*, ..., d*, *ζ_h_* ∝ *ρ*^−(^*^h−^*^1)^, $\xi_{h}\propto \rho^{h-1}\sum_{l=h}^{d}\rho^{-1}\Psi_{l}$. So for *n* large enough, *P*(*T_ext._* > *n*) ∝*ρ^n^*, but the coefficient of proportionality is not easy to calculate in practice. So we investigate other ways to calculate *G_n_*.

**Proposition 12** *For any value of R_∞_, the extinction time distribution satisfies, for n* ≥ *d,*

$$G_{n}=E(E(1_{\left\{{\tilde{\text{I}}}_{n}=0\right\}}|F_{n-d}))=E(\text{exp}(-\sum_{l=0}^{d-1}I_{n-d-l}\sum_{h=l+1}^{d}\Psi_{h})).$$

Let us notice that *G_n_* decreases (and *R_∞_* increases) as soon as at least one Ψ*_l_* increases.

In addition, using Proposition 8, we get the following result, allowing an exact iterative computation of {*G_n_*}:

**Proposition 13** *For any value of R_∞_, the distribution* {*G_n_*} *of T_ext._* *satisfies:*

$$G_{n}=\prod_{l=2}^{d}{\left[f_{n-1,l}({\text{s}}_{0})\right]}^{I_{0,l-1}}\text{exp}[-\sum_{l=1}^{d}\Psi_{l}I_{0,l}(1-{\left(\frac{G_{n-1}}{\prod_{l=2}^{d}{\left[f_{n-1,l}({\text{s}}_{0})\right]}^{I_{0,l}}}\right)}^{1/I_{0,1}})],$$

*where* **s**_0_ = (0*, ...,* 0) *and* **f***_n_*(**s**) = (*f_n,_*_1_(**s**)*, ..., f_n,d_*(**s**)) *is the offspring generating function (see Proposition 8). In the particular single-type case d* = 1, $G_{n}={\left[f(G_{n-1}^{1/I_{0}})\right]}^{I_{0}}=\text{exp}[-\Psi I_{0}(1-G_{n-1}^{1/I_{0}})]$.

Let $N:=\sum_{l=-(d-1)}^{Text.-1}I_{l}$ be the size of the tree generated by **Ĩ**_0_ (epidemic size). Let us recall that the Borel-Tanner distribution with parameter (*λ, l*) is a (*f_λ_, s^l^*) GLPD (Generalized Lagrange Probability Distribution), where *f*_λ_ (*s*) = *e*^−^*^λ^*^(1^*^−s^*^)^ is the generating function of the Poisson distribution with parameter *λ* [7, 14] (see (2)).

Let *g*(*s*) := *E*(*s^N^*) be the generating function of *N*.

**Proposition 14** *Let us assume that* $\sum_{l=1}^{d}\Psi_{l}<1$ *(subcritical case).*

1. *Let* **Ĩ**_0_ := (*I*_0_, 0, ..., 0)*. Then* $g(s)=s^{I_{0}}f_{I_{0}\sum_{l=1}^{d}\Psi_{l}}(g(s))$*, that is* $N∼\text{Borel}-\text{Tanner}(\sum_{l=1}^{d}\Psi_{l},I_{0})$:
  $$P(N=k)=\text{exp}(-k\sum_{l=1}^{d}\Psi_{l})\frac{I_{0}}{k}\frac{{\left(k\sum_{l=1}^{d}\Psi_{l}\right)}^{k-I_{0}}}{(k-I_{0})!},\;k\geq I_{0}$$
  *and* $E(N)=I_{0}{\left(1-\sum_{l=1}^{d}\Psi_{l}\right)}^{-1}$, $\text{Var}(N)=I_{0}(\sum_{l=1}^{d}\Psi_{l}){\left(1-\sum_{l=1}^{d}\Psi_{l}\right)}^{-3}$.
2. *Let* **Ĩ**_0_ := (*I*_0_, *I*_−1_, , ..., *I*_−(_*_d_*_−1)_)*. Then* $N\overset{D}{=}{⊕}_{h=1}^{d}{⊕}_{i=1}^{I_{-(h-1)}}({⊕}_{j=1}^{Y_{0,h,i}}N_{i,j}+1)$, *where* $Y_{0,h,i}∼\text{Poisson}(\sum_{l=h}^{d}\Psi_{l})$, *the* {*N_i,j_*} *are i.i.d. with* $N_{i,j}∼\text{Borel}-\text{Tanner}(\sum_{l=1}^{d}\Psi_{l},\;1)$ *and* ⊕ *means the mutual independence, that is*
  $$E(s^{N})=\prod_{h=1}^{d}s^{I_{-(h-1)}}{\left[f_{\sum_{l=h}^{d}\Psi_{l}}(g(s))\right]}^{I_{-(h-1)}},$$
  *and*
  $$\begin{array}{lll} \;\;\;E(N) & = & \sum_{h=1}^{d}I_{-(h-1)}[\sum_{l=h}^{d}\Psi_{l}{\left(1-\sum_{l=1}^{d}\Psi_{l}\right)}^{-1}+1]\\ Var(N) & = & \sum_{h=1}^{d}I_{-(h-1)}\sum_{l=h}^{d}\Psi_{l}{\left(1-\sum_{l=1}^{d}\Psi_{l}\right)}^{-3} \end{array}$$

## 6. Discussion

We presented a general class of branching processes in discrete time for modeling in a stochastic way some diseases propagation when the infected period is long respectively to the time frequency of births. However when the transitions are population dependent, the long-term prediction of these processes is an open problem in the general case. We indirectly solved this problem by studying the behavior of the limit models, as the total initial population size increases to infinity, assuming at the initial time, either a non-rare disease with a density-dependence assumption, or a rare disease.

Under the first assumption, since ${\text{lim}}_{n}{\text{lim}}_{N_{0}}{\hat{\text{P}}}_{n}|N_{n}\neq 0\overset{P}{=}{\text{lim}}_{N_{0}}{\text{lim}}_{n}{\hat{\text{P}}}_{n}|N_{n}\neq 0$, we proved, for a large *N*_0_, that the proportion of infected individuals in the whole population, ${\hat{P}}_{n}^{E}+{\hat{P}}_{n}^{I}$, behaves, as *n* → ∞, as the probability $P_{n}^{E}+P_{n}^{I}$ for an individual in an infinite population to be infected. Under the second assumption, we got a branching process on the incidence of cases which has the advantage to correspond to the usual observations, to be rigorously built, starting from a detailed multitype branching process {**N***_n_*} taking into account the different disease steps together with the population dynamic, to keep the population variability, and to belong to the simple class of multitype BGW processes for which many analytical results exist and that we used or generalized to this model. We calculated the distribution of the extinction time and the distribution of the epidemic size. Moreover we proved that the bifurcation parameter of this process was the total mean number of secondary cases who began to be generated by one case during his first time unit.

This result would validate and generalize the current use in epidemiology of the reproductive number as a bifurcation parameter [8, 17, 18], if we could establish that ${\text{lim}}_{n}{\text{lim}}_{N_{0}}{\text{N}}_{n}^{I}={\text{lim}}_{N_{0}}{\text{lim}}_{n}{\text{N}}_{n}^{I}$. We studied the left quantity, while the valid realistic quantity is in reality the right one. In fact this equality is in general not true because the bell form of an epidemic can be modelled only by a population dependent process. But for large *N*_0_, the stochastic limit model is a good approximation of the epidemic growth (in the supercritical case), or of the epidemic decay (in the subcritical case). In any case, if *N*_0_ is too small, the limit in *N*_0_ cannot be used and therefore the limit models developed here cannot be used.

Let us finally notice that we proved that in a size-dependent model on the clinical cases, then the quantity determining the extinction of the process was the total mean number of secondary cases that will be produced in the future by a case as the whole current population is infected (*R_∞_*), while the corresponding quantity in the associate deterministic model derived from the conditional expectation of the process is the total mean number of secondary cases that will be produced by a case as the whole current population is susceptible (*R*_0_), which easily leads to the extinction of the process on one hand and the persistence of the deterministic trajectory on the other hand. However, simulations of the single-type process with population-dependent offsprings described in Section 3., showed that until its extinction, the process roughly behaved as its deterministic counterpart and the extinction time strongly depends on the parameters of *R*_0_. The extinction time roughly increases as *R*_0_ increases. So the greatest difference between the behavior of this population-dependent process and its deterministic counterpart is obtained when *R*_0_ > 1 with *R*_0_ ≃ 1. This is the generalization of the difference observed between the BGW process and its deterministic counterpart, when *R*_0_(= *ρ*)= 1.
